# Retroversion of the Uterus Complicated by a Cystic Ovary and Salpingitis

**Published:** 1911-05-27

**Authors:** John Campbell

**Affiliations:** Surgeon to the Samaritan Hospital for Women, Belfast; Consulting Surgeon to the Belfast Maternity Hospital; and Clinical Lecturer and Examiner in the Queen's University of Belfast.


### Hospital Clinics.

### RETROVERSION OF THE UTERUS COMPLICATED BY A CYSTIC OVARY AND SALPINGITIS.

By JOHN CAMPBELL, M.A., M.D., LL.D., F.R.C.S. Eng., Surgeon to the Samaritan Hospital for Women, Belfast; Consulting Surgeon to the Belfast Maternity Hospital; and Clinical Lecturer and Examiner in the Queen's University of Belfast.

(Clinical Lecture delivered at the Samaritan Hospital for Women, Belfast, on December 15, 1910.)

Gentlemen,—The case we have before us is typical of its kind and is well worth careful consideration. The patient is unmarried, twenty-four years of age, and works at a sewing machine in a wareroom. Menstruation began at fourteen; has been regular every four weeks; lasts six to eight days, and is painful, the pain being mostly in the back. She has complained for two years of the following symptoms: A sensation of "burning heat" round the small of the back, not constant, worse at night and after exertion; pain in the left ovarian region, sharp and cutting in character, radiating down the thigh and across the lower abdomen, and worse on exertion. Menstruation last took place a fortnight ago and lasted six days. Micturition is painful and frequent, occurring every half-hour during the day and once or twice at night. Leucorrhœa is present. The bowels are regular. Her appetite is poor. She has no cough. Her feet and legs do not swell now, but they were badly swollen a year ago. She has had her uterus curetted in another hospital about six months ago, but has not derived any benefit.

When we examine her we find the tongue clean, the heart and lungs normal, and the abdomen presenting nothing of interest except that the right kidney is easily felt. The external genitals look normal. The hymeneal aperture is very wide, but this may be the result of previous examination and treatment. The meatus urinarius appears a little tumid. On firmly pressing the urethra against the pubic arch from above downwards, you see that we can cause a small quantity of purulent fluid to appear at the meatus. We are not now concerned with the origin of this, whether it is venereal or otherwise, but, from many careful microscopic and bacteriological examinations which I have had made in previous similar cases, we may safely conclude that this purulent urethral secretion contains staphylococci or streptococci or both, as well as less important micro-organisms. The fact which we have here to recognise is that this girl has at some time been infected as to her genital organs with pus-forming micro-organisms, and that the pelvic and bladder symptoms may depend on an extension of this infection. In passing I may remark that the presence of purulent urethral secretion is frequently associated with pelvic pain and discomfort which is extremely hard to cure; sometimes it is also associated with curious nervous or paralytic symptoms, and at other times with recurrent attacks of erythematous skin eruptions of transient duration. We have here no sign of infection of the little glands, known as Skene's glands, which run parallel with the floor of the urethra and open at the meatus urinarius. These very often retain evidence of infection even when the urethra itself has quite recovered. Nor is there anything abnormal about Bartholin's glands, though they are frequently involved in a vulvar inflammation and form retention cysts or abscesses as a result. Nothing noteworthy is felt in the vaginal walls. The cervix uteri is directed forwards. The anterior vaginal fornix is soft and yielding. The right lateral fornix is reduced in size, owing obviously to the cervix lying rather too much to the right. The left lateral fornix is correspondingly increased in size, and, on pressing up into it, a rounded firm and fixed swelling of small size is felt. This is of sufficient bulk to push the uterus a little to the right, and its presence explains the condition of both lateral vaginal fornices. The posterior fornix feels hard and firm. To the right this feeling is due to a firm fixed body, continuous with the cervix uteri and probably the retroverted uterus. To the left it is due to the presence of the small tumour previously noted when examining the left fornix. It projects back so as to be palpable in the posterior fornix as well as in the left. High up in the right of the posterior fornix you can touch a small hard tender body, probably the right ovary, lying far back in the pouch of Douglas and close to the right pelvic wall. By the bi-manual method of examination one has no difficulty in deciding that the uterus is retroverted and that there is a firm, rounded, tender and fixed tumour to the left of it. The existence of the purulent urethral secretion previously noticed gives a clue to the origin of this inflamed tumour, and shows us that we can reasonably conclude that the patient has had a vulvar inflammation which has extended by way of the vagina, uterus, and left tube as far as the left ovary. This consideration enables us to eliminate tubercular disease of the left tube and ovary which, without the presence of purulent urethral secretion, would have been probable in this case. Nothing short of removal of the left tube and ovary and ventro-suspension of the uterus will do this girl any good. Before performing this operation, however, we will have to get the urine examined bacteriologically. While the bladder symptoms may be due to the displacement of the uterus and the presence of the small tumour, still they are so pronounced that they necessitate a careful examination of the urine. In cases of purulent urethral secretion I avoid the use of the catheter, if at all possible. Here, however, the painful and frequent micturition makes it necessary for us to obtain a catheter sample of urine.

Note.—The urine was subsequently found to be normal and free from micro-organisms. Accordingly, on January 11, 1911, the abdomen was opened, the left tube and ovary removed, and the uterus suspended. The left tube and ovary were adherent and the ovary was the seat of several small cysts. The patient made a good recovery, and her uterus is now normal in position and the symptoms are gone.

Erratum.—In the article by Dr. Theodore Fisher on "Some Points in Various Affections of the Lungs" on page 182 reference is made to bronchial breathing being heard in certain conditions near the lower angle of the scapula. When heard in association with pericarditis the *left*, not the *right* side of the chest should have been mentioned.

